# Identification and characterization of a noncanonical menaquinone-linked formate dehydrogenase

**DOI:** 10.1016/j.jbc.2021.101384

**Published:** 2021-11-06

**Authors:** Rodrigo Arias-Cartín, Alexandre Uzel, Farida Seduk, Guillaume Gerbaud, Fabien Pierrel, Marianne Broc, Régine Lebrun, Bruno Guigliarelli, Axel Magalon, Stéphane Grimaldi, Anne Walburger

**Affiliations:** 1 Aix Marseille Université, CNRS, Laboratoire de Chimie Bactérienne (UMR7283), IMM, IM2B, Marseille, France; 2 Aix Marseille Université, CNRS, Laboratoire de Bioénergétique et Ingénierie des Protéines (UMR7281), IMM, IM2B, Marseille, France; 3 Grenoble Alpes Université, CNRS, Grenoble INP, TIMC, Grenoble, France; 4 Aix Marseille Université, CNRS, Plateforme Protéomique de l'IMM, IM2B Marseille Protéomique (MaP), Marseille, France

**Keywords:** bioenergetics, electron paramagnetic resonance (EPR), metalloenzyme, quinone, bacterial metabolism, BV, benzyl viologen, ECD, electrochemical detection, EPR, electron paramagnetic resonance, FDHs, formate dehydrogenases, ForC, formate oxidoreductase catalytic, ForE, formate oxidoreductase essential, Mo/W-*bis*-PGD, molybdenum/tungsten–*bis*-pyranopterin guanine dinucleotide, MSK, menasemiquinone

## Abstract

The molybdenum/tungsten—*bis*-pyranopterin guanine dinucleotide family of formate dehydrogenases (FDHs) plays roles in several metabolic pathways ranging from carbon fixation to energy harvesting because of their reaction with a wide variety of redox partners. Indeed, this metabolic plasticity results from the diverse structures, cofactor content, and substrates used by partner subunits interacting with the catalytic hub. Here, we unveiled two noncanonical FDHs in *Bacillus subtilis*, which are organized into two-subunit complexes with unique features, ForCE1 and ForCE2. We show that the formate oxidoreductase catalytic subunit interacts with an unprecedented partner subunit, formate oxidoreductase essential subunit, and that its amino acid sequence within the active site deviates from the consensus residues typically associated with FDH activity, as a histidine residue is naturally substituted with a glutamine. The formate oxidoreductase essential subunit mediates the utilization of menaquinone as an electron acceptor as shown by the formate:menadione oxidoreductase activity of both enzymes, their copurification with menaquinone, and the distinctive detection of a protein-bound neutral menasemiquinone radical by multifrequency electron paramagnetic resonance (EPR) experiments on the purified enzymes. Moreover, EPR characterization of both FDHs reveals the presence of several [Fe-S] clusters with distinct relaxation properties and a weakly anisotropic Mo(V) EPR signature, consistent with the characteristic molybdenum/bis-pyranopterin guanine dinucleotide cofactor of this enzyme family. Altogether, this work enlarges our knowledge of the FDH family by identifying a noncanonical FDH, which differs in terms of architecture, amino acid conservation around the molybdenum cofactor, and reactivity.

Formate dehydrogenases (FDHs) catalyze the oxidation of formate into CO_2_ but have also been shown to catalyze the reverse reaction in methanogenic and/or acetogenic microorganisms, namely the reduction of CO_2_ into formate (1). Metal-dependent FDHs (hereafter named FDHs) are exclusively found in prokaryotes, and 3D-structure–based phylogeny demonstrates that such enzymes were already present in the last universal common ancestor (2). In addition, paleogeochemistry suggests that in ancient times, compounds such as CO_2_ and CH_4_ were very abundant and that iron, molybdenum, and tungsten were available and in soluble forms (3, 4). Indeed, FDHs harbor molybdenum or tungsten at the active site as well as one to five iron–sulfur cluster(s) (1, 5, 6, 7, 8). The molybdenum or tungsten atom is coordinated by the dithiolene sulfurs of two organic pyranopterin guanine dinucleotides (Mo/W-*bis*-PGD) (1, 9), a cysteine or a selenocysteine and a sulfido group, all being essential for the activity (10, 11, 12). In addition, an histidine and an arginine residue located in proximity to the Mo atom (13) are strictly conserved in all FDHs described so far (Fig. 1*A*) and required for activity (14). These residues have been proposed to be involved in correct orientation or stabilization of the formate in the active site (15, 16, 17), but their role in activity is still debated. They could also act as a switch to obstruct alternatively the substrate and the product tunnels during catalysis, as suggested by the 3D structure of the FDHs from *Rhodobacter capsulatus* and *Desulfovibrio vulgaris* Hildenborough and formyl-methanofuran dehydrogenase from *Methanothermobacter wolfeii* (18, 19, 20). Altogether, there is still a need for clarifying the exact role of these conserved residues surrounding the catalytic site in FDH activity.

**Figure 1:**
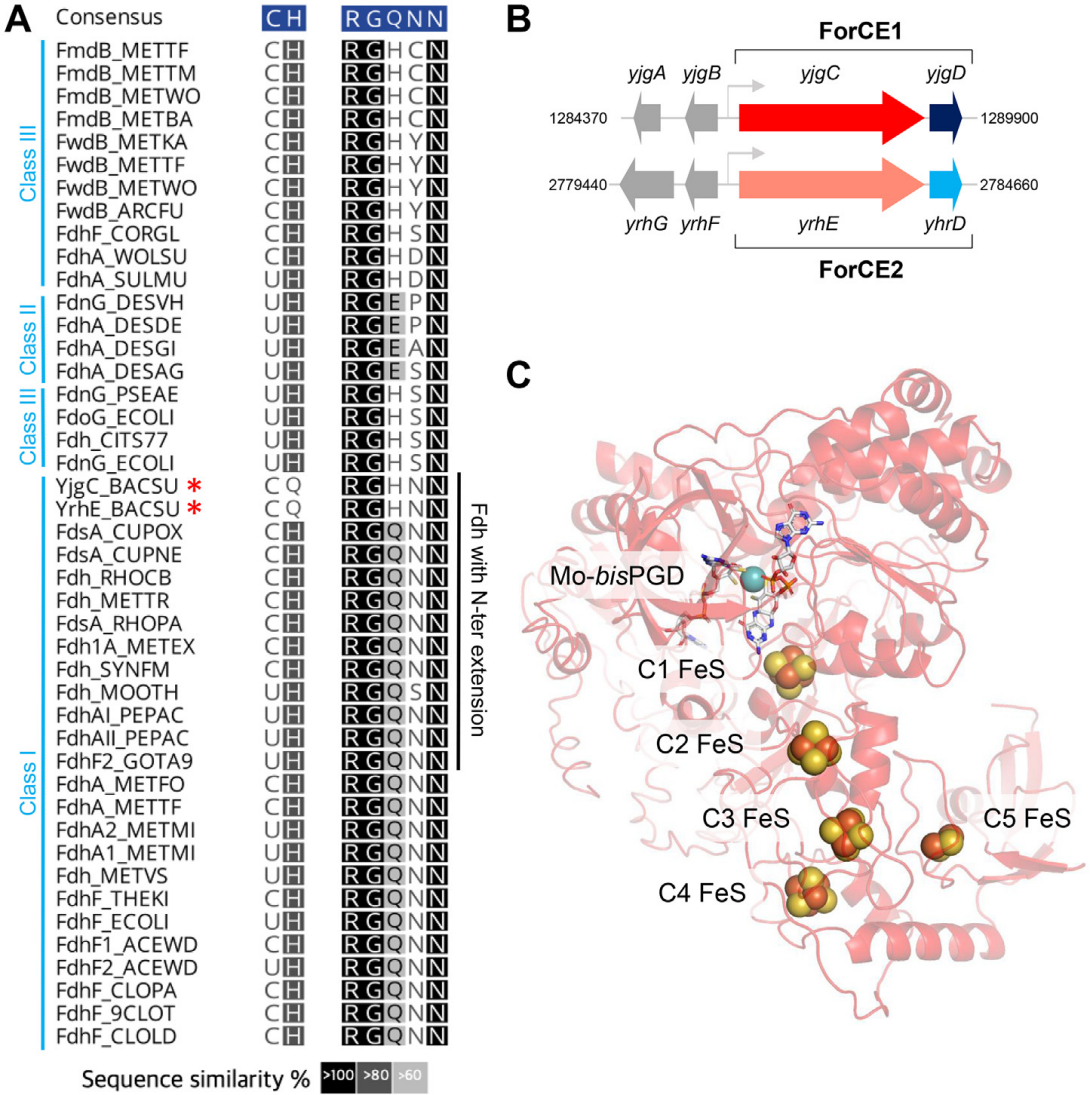
***Bacillus subtilis* genome encodes putative noncanonical FDHs.***A*, alignment of the amino acids involved in the active site of characterized FDH enzymes and the two putative FDH proteins from *B. subtilis* (*red asterisks*). Consensus sequence (*blue box*) shows which amino acids are conserved with a 50% threshold. FDHs have been organized into three classes according to the sequence and organization of their active site (Fig. S7): Class I: C/UH RGQ or C/UQ RGH, class II: C/UH RGE, and class III: C/UH RGH. Overview of the full sequence alignment is shown in Figure S1*B*. *B*, genomic organization of *yjgCD* (ForCE1) and *yrhED* (ForCE2) *in B. subtilis*. *C*, structural model of YjgC generated with I-TASSER (89) using FdsA from *R. capsulatus* (PDB ID: 6TGA). FeS clusters are named according to their proximity to the Mo cofactor in the 3D structural model. FDHs, formate dehydrogenases.

From a physiological point of view, FDHs are involved in a wide range of anaerobic metabolic pathways, such as energy harvesting and carbon fixation (6, 9). This is due to the interaction of the catalytic subunit that harbors the Mo/W-*bis*-PGD cofactor with partner subunits with the greatest diversity among the Mo/W-*bis*-PGD enzyme superfamily (9). In methanogenic archaea, FDH is part of a complex that facilitates a two-step reduction of CO_2_ to formate and then to formyl-methanofuran (20). In bacteria, the membrane-bound FDH complex couples formate oxidation to quinols production in anaerobic respiratory chains (21), and the soluble FDH from *Cupriavidus necator* and *R. capsulatus* couples formate oxidation to NADH reduction that feed the reductive pentose phosphate cycle (22, 23). To summarize, FDHs are a group of heterogenous enzymes that display different subunit composition from the single monomeric subunit FdhF from *Escherichia coli* to the six-subunit complex formyl-methanofuran dehydrogenase (13, 20), and partner subunits hold a variety of redox cofactors ([Fe-S] clusters, hemes, F_420_, FMN) that allow reaction with a wide variety of physiological redox partners, such as cytochromes, ferredoxins, NAD, and quinones (1, 5, 6, 7, 8). Altogether, identification and characterization of FDHs with variations in the amino acids surrounding the catalytic site or new subunit composition could improve our understanding of catalysis and expand the metabolic pathway repertory in which FDHs are participating.

In the archetypal gram-positive bacterium, *Bacillus subtilis*, an FDH activity has been detected in cells grown aerobically in the rich medium (24). This is intriguing as FDHs are usually part of anaerobic metabolic pathways, and *B. subtilis* is not able to produce formate (25). These observations prompted us to search for the enzyme(s) involved in this activity. Combining bioinformatics with biochemistry, enzymology, and electron paramagnetic resonance (EPR) spectroscopy, we have identified and characterized two atypical FDH enzymes in *B. subtilis* and members of a new subfamily of FDHs. Notably, formate oxidation activity is measured, whereas the conserved His residue in the active site is naturally substituted by a Gln. Moreover, both FDHs display a new architecture in which their catalytic subunit is associated with an unprecedented partner subunit that allows reactivity with menaquinone.

## Results

### The YjgC and YrhE sequences from *B. subtilis* encode putative metal-dependent FDHs

Scrutiny of the *B. subtilis* 168 genome indicates that it encodes for *yjgC* and *yrhE* genes, whose ∼110-kDa predicted proteins are paralogous with 61% of identity. Both putative oxidoreductases contain a C-terminal domain typical for Mo/W-*bis*-PGD enzymes and characterized by motifs allowing the coordination of one Mo/W-*bis*-PGD cofactor and one [4Fe-4S] cluster (Fig. S1*A* and Table S1). Furthermore, both sequences contain an N-terminal extension with binding motifs for three [4Fe-4S] clusters and one [2Fe-2S] cluster as determined by similarities (Fig. S1*A*). In particular, this region shares homologies with the N-terminal domain of the NuoG subunit of the NADH:ubiquinone oxidoreductase (*i.e.*, complex I) and the catalytic subunit of NAD-dependent [NiFe]-hydrogenases (19, 26, 27). Such domain organization is also shared with other FDHs, notably FdsA from *C. necator* and *R. capsulatus* (Figs. S1*B* and S2*A*). A close-up alignment of the active site residues with previously characterized FDH sequences and YjgC and YrhE sequences is shown in Figure 1*A*. Whereas the typical metal Cys ligand and the strictly conserved Arg residue are present, the highly conserved His residue is substituted by a Gln in YjgC and YrhE. Phylogenetic analysis identified a large group of YjgC/YrhE-related sequences with the similar substitution in several bacterial and archaeal phyla, which are distinct from FdsA-related sequences (Fig. S2*A*). The presence of the Gln next to this Cys essential residue in the primary sequence questions the activity of these enzymes as FDHs. However, for the evidence that *B. subtilis* possesses at least one FDH is the existence of a putative *fdhD* gene encoding a sulfurtransferase crucial for FDH activity (12). In the *B. subtilis* genome, the *fdhD* gene is distant from the *yjgC* and *yrhE* loci (53′ *versus* 18′ and 39′, respectively). Interestingly, the *yjgC* gene is predicted to be organized in operon with *yjgD* as *yjgC* and *yjgD* are coexpressed with a positive correlation of 0.98 (28, 29). The same holds true for the gene *yrhE* predicted to be in operon with *yhrD* as both are adjacent and coexpressed with a positive correlation of 0.99 (Fig. 1*B*). The *yjgD* and *yrhD* genes encode 21-kDa and 17-kDa polypeptides, with weak sequence overall identity (20%), and contain a DUF1641 domain of 38 amino acids of unknown function (PFAM PF07849). While YjgC/YrhE are related to FdsA, as illustrated by the YjgC structural model based on the 3D structure of *R. capsulatus* FdsA (Fig. 1*C*) and the phylogenic analysis (Figs. 1*A* and S1), YjgD and YrhD are distinct from FdsD or any of the other partner subunits previously characterized for an FDH, the complex I and [Ni-Fe] hydrogenases (6). In addition, YjgD and YrhD are predicted to be cofactor-less and have a distinct sequence from the FdsC/FdhD sulfurtransferase or any specific chaperone involved in Mo-*bis*-PGD maturation (30, 31). Altogether, these *in silico* analyses suggest that YjgC and YrhE are the enzymes involved in the FDH activity previously detected in *B. subtilis* (24) and question the role of YjgD and YrhD.

### The YjgCD and YrhED are protein complexes with formate-oxidizing activity

**Figure 2:**
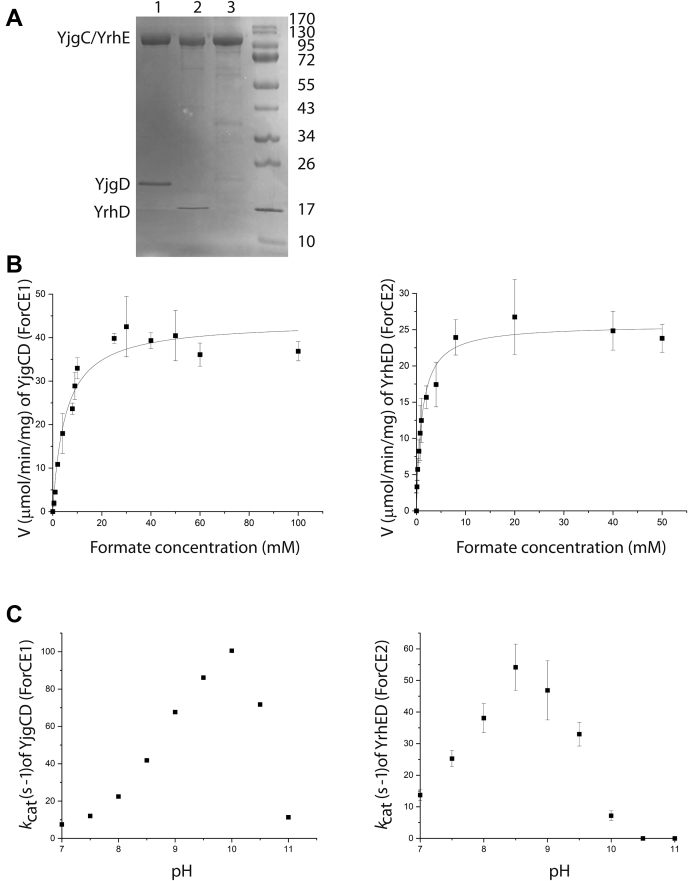
**YjgCD (ForCE1) and YrhED (ForCE2) are formate-oxidizing enzymes.***A*, purified proteins are analyzed on Coomassie Brilliant Blue–stained 15% SDS-polyacrylamide gel. Molecular standards are indicated in kilodalton. *Lane 1*: YjgC N-terminus 6his-tagged copurifies with YjgD; *lane 2*: YrhE N-terminus 6his-tagged copurifies with YrhD; *lane 3*: YjgC C-terminus 8his-tagged was purified without YjgD. *B*, Michaelis–Menten plot for the formate:benzyl viologen oxidoreductase reaction of YjgCD (ForCE1) at pH 10 and YrhED (ForCE2) at pH 8.6. *C*, pH dependence of *k*_cat_ (s^−1^) for YjgCD (ForCE1) and YrhED (ForCE2).

To test the activity of YjgC and YrhE as FDHs and the importance of YjgD and YrhD, the corresponding proteins were purified. First, the *yjgCD* and *yrhED* operons were expressed in *B. subtilis* under the control of an inducible promoter and a 6-histidine tag was added at the N terminus of the YjgC and YrhE proteins. Affinity purification of these proteins showed that YjgC and YrhE copurified with YjgD and YrhD, respectively, as identified by SDS-PAGE and MS analysis (Fig. 2*A*, Tables S2 and S3). MS data are available via ProteomeXchange with identifier PXD028742. These results suggest that proteins YjgD and YrhD interact physically with YjgC and YrhE, respectively. A full steady-state enzyme kinetic analysis of formate oxidation by YjgCD and YrhED using benzyl viologen (BV) as an artificial electron acceptor is shown in Figure 2*B*. Using the Michaelis–Menten plot, a *k*_cat_ of 96 s^−1^ was measured for the YjgCD heterodimer (MW = 132 kDa) and a *k*_cat_ of 56 s^−1^ for the YhrED heterodimer (MW = 125 kDa) (Table 1). Both values are in the same range as the ones determined for FDH of *R. capsulatus* (36 s^−1^) and *C. necator* (201 s^−1^) (22, 26). Apparent *K*_M_^formate^ of 5 mM for YjgCD and 1 mM for YrhED are larger than the values of 0.281 mM and 0.31 mM determined for FDH of *R. capsulatus* and *C. necator*, respectively. As observed for other FDHs (22, 32, 33), both enzyme preparations exhibit a basic pH optimum, pH 10 for the YjgCD complex and pH 8.5 for YrhED (Fig. 2*C*). In conclusion, the YjgCD and YrhED are protein complexes with formate-oxidizing activity with kinetic parameters comparable with other FDHs despite the fact that the amino acid sequence within the active site deviates from the consensus residues typically associated with FDH activity. Henceforth, the YjgC subunit was renamed ForC for formate oxidoreductase catalytic subunit and the YjgD subunit ForE for formate oxidoreductase essential subunit for reasons that will be presented at the end of the Results section. As *B. subtilis* encodes two FDHs, YjgCD was renamed ForCE1 and YrhED was renamed ForCE2.

**Table 1:** Enzymatic catalytic constants for formate oxidation by YjgCD, YjgC, and YrhED using either benzyl viologen (top) or menadione (bottom) as electron acceptors

FDH	*K*_M_^formate^ (mM)	*k*_cat_ (s^−1^)	*V*_max_ (U)	pH
YjgCD/ForCE1	5.1 ± 0.9	96.1 ± 4	43.7 ± 1.9	10
YrhED/ForCE2	1.1 ± 0.1	55.8 ± 1.6	26.6 ± 0.8	8.6
YjgC/ForC1	ND	2.13 ± 0.05	1.16 ± 0.03	10


	*K*_M_^menadione^ (mM)	*k*_cat_ (s^−1^)	*V*_max_ (U)	pH
YjgCD/ForCE1	0.025 ± 0.003	16.5 ± 0.6	7.5 ± 0.3	9
YrhED/ForCE2	0.023 ± 0.005	26.4 ± 1.8	12.6 ± 0.9	9
YjgC/ForC1	ND	0	0	9

Abbreviation: ND, not determined.

### EPR analysis of ForCE1 and ForCE2

**Figure 3:**
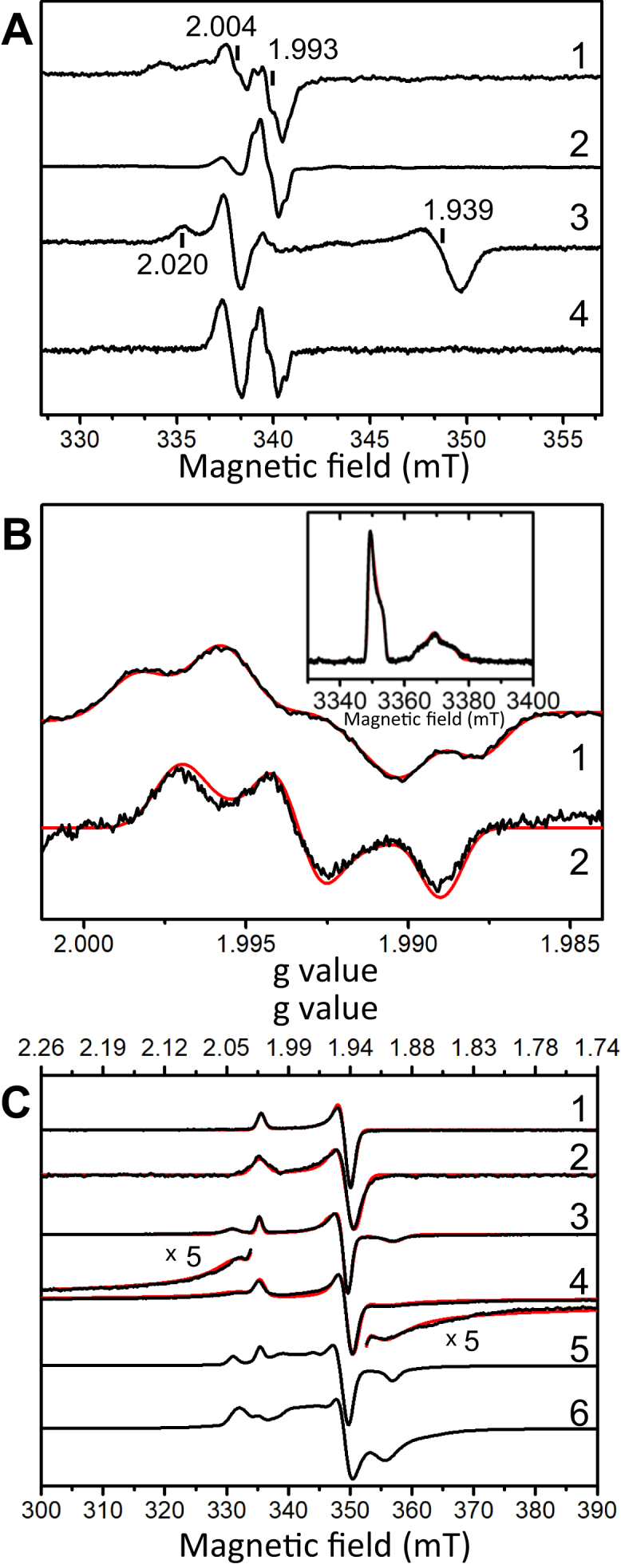
**EPR characterization of the cofactors in ForCE1 and ForCE2.***A*, air-oxidized (1 and 2) and formate-reduced (3 and 4) ForCE1 (1 and 3) and ForCE2 (2 and 4) collected at 50 K (1) or 70 K (2–4). Other experimental conditions were as follows: microwave power, 4 mW, field modulation amplitude, 0.4 mT (1 and 2) or 0.2 mT (3 and 4) at 100 kHz, and microwave frequency, ∼9.48 GHz. *Vertical lines* indicate remarkable g values given in the figure and discussed in the main text. *B*, continuous wave Mo(V) EPR spectra in air-oxidized ForCE2 samples measured at X band (9.47864 GHz) (1) and Q band (34.1187 GHz) (2) frequencies. Other experimental conditions were as follows: temperature, 70 K (1) and 50 K (2), microwave power, 1 mW (1) and 0.1 mW (2), and field modulation amplitude, 0.1 mT (1) and 0.5 mT (2) at 100 kHz. The inset shows the echo-detected field-swept EPR spectrum measured at 50 K at W-band (94.0014 GHz) frequency. Other experimental conditions are as follows: microwave pulse lengths, 20 ns and 40 ns for π/2 and π pulses, respectively. *C*, EPR spectra of dithionite-reduced ForCE1 (1, 3, and 5) and ForCE2 (2, 4, and 6) measured at 100 K (1 and 2), 50 K (3 and 4), or 15 K (5 and 6). Other experimental conditions were as follows: microwave power, 1 mW (1) or 10 mW (2–6), modulation amplitude 1 mT (1, 2, 4–6) or 0.4 mT (3) at 100 kHz, microwave frequency, ∼ 9.48 GHz. Spectral simulations are displayed as *red traces* superimposed to the experimental spectra and have been performed using the parameters given in the main text and supporting information (Table S8).

To characterize the cofactor content of the two enzymes, we next examined the EPR properties of purified ForCE1 and ForCE2 enzymes, either in their air-oxidized state or after incubation with a large excess of sodium formate or sodium dithionite. When measured in the 50- to 70-K temperature range, the air-oxidized enzymes mainly display two essentially isotropic and partially overlapping EPR signals originating from two slow-relaxing S = ½ species with g-values centered at 2.004 and 1.993. Their relative intensities are different in the two enzymes (Fig. 3*A*, traces 1 and 2). In a first step, the g = 1.993 signal that exhibits a partially resolved structure in both enzymes was investigated in detail. Optimally detected in the air-oxidized ForCE2 sample, this signal was further characterized by performing echo-detected field-swept experiments at W-band (∼94 GHz) or continuous wave EPR measurements at Q-band (∼34 GHz) (Fig. 3*B*, inset and trace 2). At both frequencies, the g = 1.993 signal is well isolated and displays a rhombic shape well reproduced by considering a single species with g-values g_1__,__2,3_ = 1.9971, 1.9933, and 1.9890. Whereas this model is unable to account for the resolved structure on the X-band EPR spectrum, the addition of a strongly coupled proton with nearly isotropic ^1^H hyperfine coupling tensor having principal values A_1,2,3_ = 13, 13, and 11 MHz allows reproducing the observed splittings (Fig. 3*B*, trace 1). Eventually, the multifrequency EPR spectra are well simulated using the same set of g- and A-tensors as given above (Fig. 3*B*). The signal-giving species displays g-values fully consistent with those of a 4 d^1^ Mo(V) ion with *bis*-pterin coordination (34). Notably, the corresponding g-tensor anisotropy (g_1_ – g_3_ ∼ 0.008) is unusually low and most closely resembles that of the slow-type Mo(V) signal *Desulfovibrio alaskensis* NCIMB 13491 FDH (g_1_ – g_3_ ∼ 0.012) (35). Moreover, the Mo(V) g-values in ForCE2 belong clearly to the correlation found for the variations of the g-tensor of Mo(V) species with a six-sulfur coordination (9, 34, 36) (Fig. S3). Finally, the magnitude of the proton hyperfine coupling tensor is comparable with that resolved on the Mo(V) EPR signal detected in *C. necator* FDH and assigned to a molybdenum-SH group (26). Therefore, the g = 1.993 signal similarly detected in ForCE1 and ForCE2 is unambiguously assigned to a Mo(V) species generated at their active site.

Interestingly, incubation of the enzymes with a large excess of sodium formate leads to an increase of the g = 2.004 signal relative to that of the Mo(V) species (Fig. 3*A*, traces 3 and 4). In addition, an axial signal with g-values 2.020 and 1.939 is detected in the ForCE1 spectrum measured at 50 K (Fig. 3*A*, trace 3). These observations are explained by partial reduction of the enzymes by sodium formate and are consistent with the fact that the latter is the physiological substrate. The same axial signal is observed on the EPR spectrum of the dithionite-reduced samples of ForCE1 and ForCE2 measured at 100 K, whereas the Mo(V) signal and the g = 2.004 species are no longer detected, consistent with further reduction of the latter and of the Mo(V) into the EPR-silent Mo(IV) state in these samples (Fig. 3*C*, traces 1 and 2). The slow relaxation properties of this axial signal and its g-values are typical for S = ½ [2Fe-2S]^1+^ clusters (37) and very similar to those detected in related FDHs from *Clostridium thermoaceticum*, *Methylosinus trichosporium*, or *C. necator* (26, 38, 39, 40). Upon lowering the temperature to 50 K, new signals are detected peaking at g = 2.047 and 1.897 in ForCE1 (Fig. 3*C*, trace 3), and g = 2.030 and 1.912 in ForCE2 (Fig. 3*C*, trace 4). These are tentatively assigned to the g_1_ and g_3_ values of an additional slow relaxing S = ½ [4Fe-4S]^1+^ cluster. The corresponding intermediate g_2_ value is estimated by numerical simulations (shown as red solid lines in Fig. 3*C*, traces 3 and 4), yielding to g_1__,__2__,__3_ = 2.047, 1.948, 1.897 and 2.030, 1.940, 1.912 for this cluster in ForCE1 and ForCE2, respectively. Further lowering the temperature to 15 K leads to the appearance of additional overlapping signals peaking at 1.99 and 1.96, which most likely arise from an additional fast-relaxing [4Fe-4S]^1+^ center (Fig. 3*C*, traces 5 and 6). Being the sole EPR active center detected at 100 K, the [2Fe-2S] cluster was used as an internal reference for spin quantitation measurements. Double integration of the EPR spectrum of a dithionite-reduced ForCE1 sample buffered at pH 7.5 and measured at 15 K in nonsaturating conditions (*i.e.*, 0.1 mW) indicated that the detected signals contribute to about 4.7 ±0.4 spins/ForCE1, assuming one [2Fe-2S] center per enzyme.

Overall, the present EPR analysis allows identifying the signature of several metal centers in ForCE1 and ForCE2, including that of their Mo-*bis*-PGD cofactor, a [2Fe-2S] cluster and at least two other [4Fe-4S] centers with distinct relaxation properties. Importantly, the detected species have quite similar EPR signatures in both enzymes in agreement with the high identity percentage between ForC1 and ForC2 subunits.

### A menasemiquinone radical stabilized within ForCE1 and ForCE2

**Figure 4:**
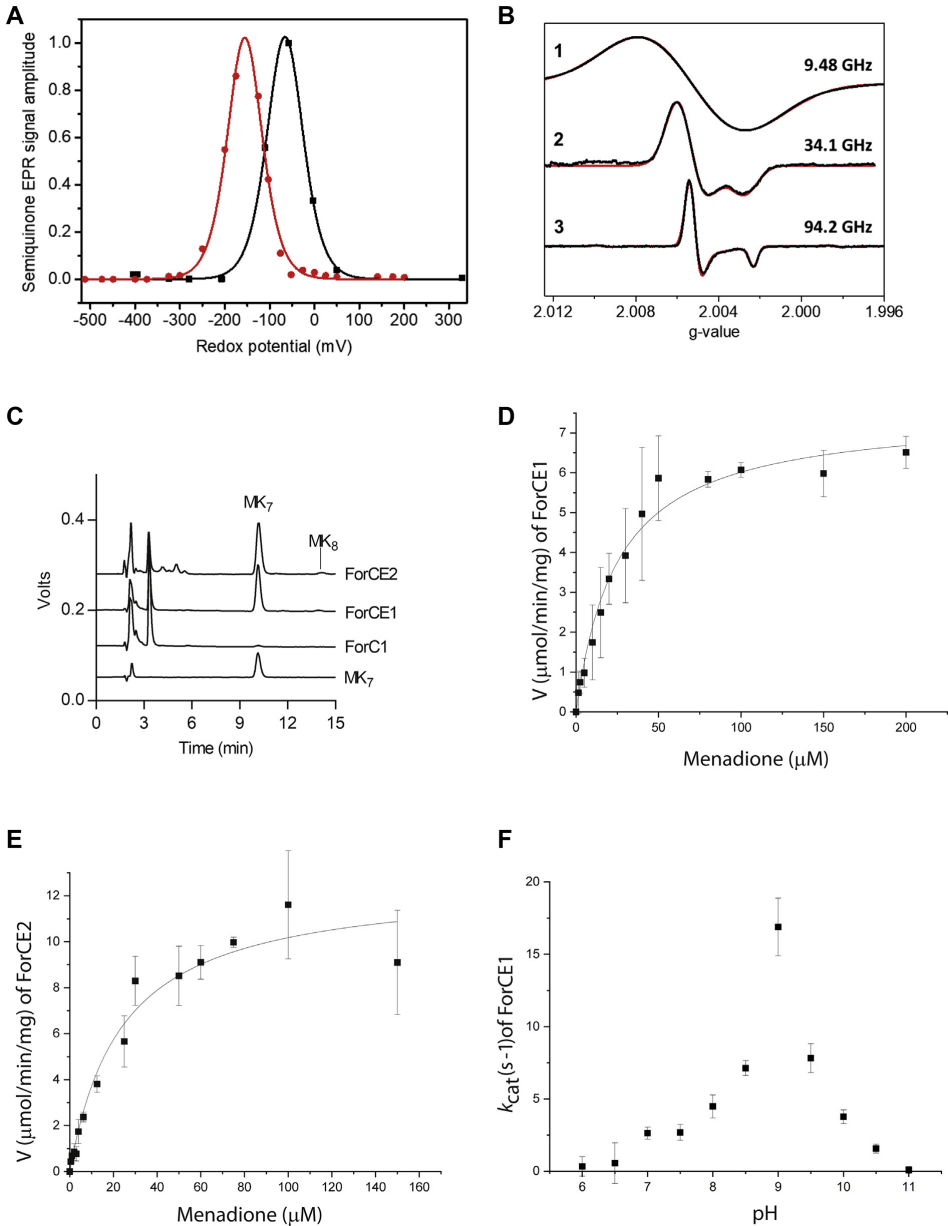
**ForCE1 and ForCE2 bind MK-7 as an electron sink.***A*, normalized redox titration curves of the MSK radical in ForCE1 buffered at pH = 6 (*black*) or 7.5 (*red*). Experimental points (*filled squares* or *filled circles*) have been fitted to the theoretical curve as described in the Experimental procedures section. Experimental conditions were as follows: temperature, 50 K, microwave power, 10 mW, and modulation amplitude, 0.5 mT (pH 7.5) or 1 mT (pH 6) at 100 kHz. *B*, g-scale representation of the cw EPR spectra (*black traces*) and their simulation (*red traces*) of the MSK^.^ radical in ForCE1 buffered at pH = 6 and measured at X band (9.4812 GHz) (1), Q band (34.094 GHz) (2), and W band (94.244 GHz) (3). Spectra were simulated using the same rhombic g-tensor with principal values g_1_,_2_,_3_ = 2.0054, 2.0051, and 2.0023 and the linewidths given in supporting information (Table S8). Experimental conditions were as follows: temperature, 50 K, microwave power, 4 mW (1), 0.1 mW (2), or 0.05 mW (3), and field modulation amplitude at 100 kHz, 0.4 mT (1 and 3) or 0.5 mT (2). *C*, ForCE1 and ForCE2, but not ForC1, bind large quantities of MK-7. HPLC-ECD analysis of the MK-7 standard (15 pmol) and of lipid extracts corresponding to ∼40 pmol of purified proteins. *D*, Michaelis–Menten plot for the formate:menadione oxidoreduction reaction of ForCE1 at pH 9. *E*, Michaelis–Menten plot for the formate:menadione oxidoreduction reaction of ForCE2 at pH 9. *F*, pH dependence of *k*_cat_ (s^−1^) for ForCE1.

An unexpected outcome of the EPR analysis presented above is the detection of an intense isotropic radical signal at g = 2.004 in ForCE1 and ForCE2 after incubation with sodium formate (Fig. 3*A*, traces 3 and 4). The radical peak-to-peak linewidth (∼0.88 mT) is similar to that typically observed for protein-bound isoprenoid quinones (41, 42). To further identify its origin, its redox properties were investigated using EPR-monitored redox titration experiments performed on purified ForCE1 buffered at pH 6.0 and 7.5. The EPR amplitude of the radical measured against ambient redox potential changes according to a bell-shaped curve as expected for quinone species undergoing two successive one-electron reduction steps between the fully oxidized quinone and the fully reduced quinol state with an EPR-active semiquinone intermediate (Fig. 4*A*) (42, 43, 44, 45, 46). When the data were fitted using theoretical curves based on Equation 1, midpoint potential values (E_1_, E_2_) = (−42 mV, −90 mV) and (−136, −175 mV) were obtained at pH 6 and 7.5, yielding two-electron midpoint potential values E_m_ of −66 mV and −156 mV, respectively. Such values are consistent with those of low-potential menaquinones (E_m, pH 7.5_ MK/MKH_2_ = −100 ± 10 mV) (47) such as MK-7, the major quinone present in *B. subtilis* membranes (48). The −60 mV/pH unit dependence of E_m_ in the investigated pH range indicates a 2H^+^/2e^−^ reaction as measured for semiquinone radicals stabilized in exchangeable Q sites (43, 44, 45). In addition, the similar −60 mV/pH unit dependence of both E_1_ and E_2_ indicates that the neutral menasemiquinone (MSK) form (*i.e.*, MSKH^•^) is stabilized in this pH range. The maximum concentration of the radical was estimated to be 0.5 MSK/ForCE1 from double integration of its EPR spectrum measured under nonsaturating conditions and using the [2Fe-2S] cluster as internal standard, assuming one [2Fe-2S]/enzyme.

The chemical nature of the radical was further confirmed by performing EPR experiments at higher microwave frequencies, as large unresolved hyperfine couplings typically obscure the g anisotropy of radicals measured at standard X-band frequencies (9–10 GHz) (49). Whereas the g tensor anisotropy of the radical in ForCE1 is only partially resolved at the Q band (∼34 GHz), its g_3_ component is clearly resolved at the W band (∼94 GHz), the overall line shape remaining predominantly axial (Fig. 4*B*). Such features are again expected for protein-bound isoprenoid semiquinones (42, 50). In addition, EPR spectra recorded at X, Q, and W bands are well simulated using the same rhombic g-tensor with principal values g_1__,__2__,__3_ = 2.0054, 2.0051, 2.0023. These are very close to those measured for other protein-bound MSK such as in the Q_D_ site of *E. coli* nitrate reductase (42, 46) (Fig. S4) or in the Q_A_ site of Zn-substituted reaction center from *Blastochloris viridis* (41). Therefore, the EPR signal of the radical detected in ForCE1 can be assigned to a neutral MSK radical bound to the enzyme based on its EPR properties evaluated at several microwave frequencies as well as on its redox characteristics. This assignment also holds for the radical species detected in ForCE2, which exhibits similar g-values, as concluded from the comparative analysis of the W band EPR spectra of the radical measured in ForCE1 and in ForCE2 (Fig. S4).

In addition, our data reveal that the detected MSK is highly stabilized in ForCE1 in the pH range from 6.0 to 7.5. The magnitude of the MSK stability constant is virtually independent of pH (K_S, pH = 6.0_ ∼ 7, K_S, pH = 7.5_ ∼ 5) and much larger than that expected for the unbound species, that is, K_S, free_ ∼ 10^−15^. Hence, the calculated occupancy level R_occ_ of the ForCE1 Q-site that corresponds to the fraction of the site occupied by MK, MSK, or MKH_2_ (irrespective of their protonation state) is close to unity at both pH values (R_occ_ ∼ 0.8 at pH = 6.0 and ∼ 0.9 at pH 7.5).

We confirmed the nature of the MK bound in purified ForCE1 and ForCE2 by analyzing lipid extracts with HPLC coupled to electrochemical detection and MS (HPLC-ECD-MS) (Fig. 4*C*). Lipid extracts from ForCE1 and ForCE2 show a predominant peak at 9.8 min, which coincides with the MK-7 standard. MS confirmed the identity of MK-7 (Fig. S6*A*). A minor peak of MK-8 was also detected (Fig. 4*C*), as supported by MS analysis (Fig. S6*B*). While the ForCE1-MK-7 ratio inferred by HPLC-ECD analysis shows a stoichiometric value of 1:0.87, the ForCE2-MK-7 ratio is above 1:1 stoichiometry (1:1.63) (Table S5). Nevertheless, the ForCE1-MK-7 ratio is in agreement with the amount of one MK-7 per complex quantified by EPR analysis.

### ForCE1 and ForCE2 reduce MK-7 analogs

Having demonstrated that ForCE1 and ForCE2 enzymes are able to stabilize an MSK radical, we hypothesized that MK-7 is the electron acceptor during formate oxidation in *B. subtilis*. The enzymatic activity of purified ForCE1 and ForCE2 was analyzed in the presence of formate and menadione, a structural analog of MK-7. Full steady-state enzyme kinetic analyses are shown in Figure 4, *D* and *E*. To model experimental data, we used a Michaelis–Menten plot and found similar apparent low *K*_M_^menadione^ values for both enzymes (25 μM for ForCE1 and 23 μM for ForCE2) and a *k*_cat_ of 16.5 and 26.4 s^−1^ for ForCE1 and ForCE2, respectively (Table 1). The pH dependence of the formate:menadione oxidoreductase activity was determined and showed a pH optimum at ≈ 9 for both enzymes (Figs. 4*F* and S5). Altogether, our *in vitro* kinetic analyses show that ForCE1 and ForCE2 are able to couple formate oxidation to menadione reduction, and we propose that MK-7 is the physiological electron acceptor during formate oxidation.

### ForE1 is an essential subunit for formate oxidoreductase activity of ForC1

To assess the importance of ForE1 or ForE2 in FDH activity, the *yjgC* (*forC1*) and *yrhE* (*forC2*) genes were expressed under the control of an inducible promoter in absence of *yjgD* (*forE1*) and *yrhD* (*forE2*), respectively. Despite many attempts, we were unable to purify the ForC2 subunit His-tagged in C-terminal when produced without ForE2. On the contrary, we were able to purify ForC1 His-tagged in C-terminal (Fig. 2*A*, Table S4). This protein was able to oxidize formate with BV as an artificial electron acceptor but at a residual *k*_cat_ = 2 s^−1^ compared with 96 s^−1^ for the ForCE1 complex (Table 1). HPLC-ECD analysis of ForC1 showed that very low quantities of MK-7 are associated with ForC1, as compared with ForCE1 (Fig. 4*C*, Table S5), and ForC1 is devoid of formate:menadione oxidoreductase activity (Table 1). Thus, the ForE1 subunit is required for formate:menadione oxidoreductase activity of ForC1 and most likely for MK-7 binding. Altogether, these results demonstrate the essential role of ForE1 in ForCE1 activity.

## Discussion

FDHs have been widely studied as they reversibly catalyze formate oxidation into CO_2_, CO_2_ reduction being of high biotechnological interest. In contrast to other members of the Mo/W-*bis*-PGD enzyme superfamily, FDHs exhibit a plethora of quaternary structure, subunit, and redox center composition and cellular localization allowing their integration in several anaerobic metabolic pathways as reviewed (5, 7, 9, 51). Here, we report on the identification, isolation, and characterization of two atypical FDHs, ForCE1 and ForCE2, in the aerobic soil bacterium, *B. subtilis*. In contrast with other characterized FDHs, ForCEs (i) have a unique subunit composition, (ii) display modification of the hitherto conserved amino acid residues surrounding the metal active site, and (iii) bind MK-7 while being cytosolic enzymes as shown by the homologous expression system reported here. Notably, ForCE1 and ForCE2 have each a Mo-*bis*-PGD cofactor and bear up to 5 [Fe-S] clusters as determined by EPR spectroscopy. In addition, both enzymes are able to perform formate:menadione oxidoreduction and have the ability to strongly bind MK-7 as indicated by HPLC-ECD-MS analysis, which leads to the detection of an isotropic radical signal by EPR spectroscopy, assigned to an MSK signal.

Multimeric FDHs work in concert with other subunits to oxidize formate or receive electrons from a donor to reduce CO_2_. In other terms, partner subunits allow FDH integration into a specific metabolic pathway. Gene(s) encoding partner subunit(s) are organized into operons with the gene encoding the catalytic subunit. In FDHs from *B. subtilis*, genes encoding catalytic and partner subunits are organized into *forCE1* and *forCE2* operons. Our results have shown that purified ForC1 produced in absence of ForE1 binds weakly to MK-7 and that it has lost formate:menadione oxidoreductase activity. Accordingly, we suggest that ForE is the partner subunit that allows coupling formate oxidation to quinone reduction. Such reaction has been demonstrated for the respiratory FDH complex exemplified in *E. coli* (21). However, the partner subunits FdnHI are very distinct from ForE at the sequence level. The Mo-*bis*-PGD containing the FdnG subunit is the formate oxidation site connected through a 5 [Fe-S] cluster electron wire (in FdnGH) to the quinone reduction site in the transmembrane cytochrome FdnI. In contrast, the ForC subunit harbors the Mo-*bis*-PGD cofactor formate oxidation site as well as 5 [Fe-S] clusters in the same polypeptide and the menaquinone reduction site hypothetically located at the ForCE interface. It must be noted that ForCE sequences are not predicted to contain any transmembrane segment, neither hemes, as confirmed by EPR analysis of ForCE1 and ForCE2. Therefore, we conclude that the way the formate:menaquinone oxidoreduction occurs into ForCEs is unprecedented.

The ForC catalytic subunit presents high sequence similarity with FdsA from *R. capsulatus* and *C. necator* (Fig. S1) and a ForC1 structural model using the former has been built (Fig. 1*C*). In this model, electrons are logically shuttled from the Mo-*bis*-PGD catalytic site along the [Fe-S] clusters (C1 to C4/C5) to the physiological electron acceptor. We hypothesize that MK-7 binds to the N-terminal region of ForC1 in such a way that the distance between the MK-7 and one of the two most N-terminus [Fe-S] clusters, namely the [4Fe-4S] (C4) or the [2Fe-2S] (C5) cluster (Fig. 1*C*), is compatible with physiological electron transfer (52). It is interesting to note that EPR-monitored redox titration experiments performed on purified ForCE1 buffered at pH 6.0 and 7.5 have provided values of E_m_ (MK/MKH_2_) about 55 mV lower than that of the menaquinone pool. Such a shift can be explained by an ∼ 80-fold tighter binding of oxidized MK than MKH_2_ to the ForCE1 Q-site, which would be expected for a quinone reduction site in which binding of the substrate (menaquinone) is favored over that of the product (menaquinol). This situation is very similar to that described for the plastosemiquinone bound to the quinone reduction site QB in photosystem II (Δ = −50 mV) (53). In addition, similar values have been measured for the demethylmenasemiquinone stabilized at the quinol oxidation site of the membrane-bound nitrate reductase from *E. coli* (Δ = −30 or −80 mV) (54), and tighter binding of ubiquinol than ubiquinone has been discussed in the quinone reduction site of the *bc*_1_ complex from yeast (Δ ∼ +24 to +54 mV) (55) or from *Rhodobacter sphaeroides* (Δ ∼ +60 mV) (56).

Within the enzymatic complex, the ForE1 subunit could help stabilize menaquinone binding by providing a structural interface with ForC1 as in its absence, the amount of copurified MK-7 drops dramatically to 1.6%. Moreover, ForE1 may participate to the structural integrity of ForC1 as its formate:BV oxidoreductase activity dropped by 50-fold when produced and purified in the absence of ForE1. Using several techniques, we have shown that MK-7 copurified with ForCE1 in a molar ratio (1:0.87). As discussed above, ForCE enzymes are not predicted to contain any transmembrane helices and are purified without detergents. Nevertheless, they bind menadione with an apparent affinity (*K*_M_^menadione^ = 20 μM) in the same range as the membrane integral nitrate reductase A from *E. coli* and the peripheral type-II NADH:quinone oxidoreductase from *Caldalkalibacillus thermarum* (57, 58). Finally, ForCE1 is also able to bind MK-8 at a low but significant ratio, consistent with low MK-8 abundance in *B. subtilis* membrane (48). To recruit menaquinone, it is reasonable to assume that ForCE contacts the membrane. Even if ForCE1 and ForCE2 have been purified without detergents, they could loosely bind the cytoplasmic side of the membrane through amphipathic helices similarly to the peripheral NdhII from *C. thermarum* that couples NADH oxidation to menaquinone reduction (59). Evaluation of this hypothesis is the object of undergoing studies in our laboratory.

ForC1 and ForC2 from *B. subtilis* are distinct from other members of the FDH family notably because of the residues at vicinity of the Mo atom such as the natural substitution of the strictly conserved His residue adjacent to Cys or Secys (C/UH) by a Gln (C/UQ) (Figs. 1*A* and S2*A*). This change from a positively charged and polar amino acid (His) by a polar one (Gln) is intriguing and prompted us to examine the active site region by sequence alignment and examination of the 3D structures of FDHs (Fig. S7). This scrutinous analysis allowed us to pinpoint an His residue located beside the strictly conserved Arg active residue and whose function may be to preserve at least one positive charge in close proximity to the active site (Fig. S7*A*). Furthermore, this analysis sheds light into the systematic occurrence of at least one His residue close to the active site with the occurrence of a Gln residue (Class I: C/UH RGQ or C/UQ RGH as in ForC, Fig. S7, *A–C*), a Glu residue (Class II: C/UH RGE, Fig. S7, *D* and *E*), or an His residue (class III: C/UH RGH, Fig. S7, *F* and *G*). As mentioned before, the importance of these residues and their mechanistic role at the active site are still unknown, and this will be investigated in detail in forthcoming studies.

Phylogenetic analysis indicates that the ForC proteins of *B. subtilis* are the first characterized representatives of a new subfamily of FDHs with a CQ RGH motif. Indeed, using the ForC1 sequence as bait, 377 sequences with an N-terminal extension allowing the coordination of 4 [Fe-S] clusters were identified. These sequences belong to the previously mentioned classes I and III. Strikingly, the 176 sequences with the CQ RGH catalytic motif (Fig. S2*A* sequences shown in blue) cluster distinctly from the 201 sequences with the CH RGQ/H catalytic motif (Fig. S2*A* sequences shown in gray). The 176 sequences are mainly found in Firmicutes, but some representatives are found in other bacterial phyla (Proteobacteria, Actinobacteria, Deinococcus, Acidobacteria, and Planctomycetes) or in Archaea (Fig. S2*B*). Furthermore, the vast majority of sequences with the CQ RGH motif are synteny encoded with a gene encoding a protein with a DUF1641 domain, presumably a homolog of ForE.

While the *forC1* and *forC2* genes are paralogous and proteins have 61 % of identity, ForE1 and ForE2 share weak sequence identity (20%). However, both enzymatic complexes achieve the same reaction with comparable kinetic behavior and most likely interact loosely with the membrane to recruit menaquinone. A notable difference between both complexes resides in the way the corresponding operons are regulated. While genes encoding FDHs are typically regulated by anaerobiosis, fermentation, and formate induction (60, 61), *forCE1* and *forCE2* do not seem expressed under these metabolic conditions (24, 28) and are upregulated by different environmental and cellular cues. Notably, the *forCE1* operon is part of the SigB-dependent general stress regulon and is upregulated in swarming conditions, in the presence of high salt or ethanol concentrations (28, 62, 63). On the other hand, the *forCE2* transcription is upregulated after germination and in exponential growth and downregulated by the ResED two-component system that activates genes in oxygen-limited conditions (28, 64).

Altogether, our results highlight the originality of these noncanonical FDHs, expand our knowledge on the FDHs' building blocks, and pave the way for developing new biocatalysts for CO_2_ reduction.

## Experimental procedures

### Bacterial strains, media, and culture conditions

*B. subtilis* 168 (*trpC2*) strain and derivatives are described in Table S6. Genomic integration of reporter sequences was achieved using the pSPH1/pSPH2 plasmids (obtained by Dr Henrik Strahl von Schulten) by transformation at the *amyE* locus (65). Target integration was confirmed by amylase sensitivity. Chromosomic inactivation of target genes was obtained by transformation of gDNA from KO strains (source BGSC). Clones were verified by PCR. For routine growth, cells were propagated in LB medium (tryptone, 10 g/l, yeast extract, 5 g/l, NaCl, 5 g/l). When necessary, antibiotics were used at the following concentrations: spectinomycin (100 μg ml^−1^), kanamycin (10 μg ml^−1^), and erythromycin (1.25 μg ml^−1^).

For overproduction of recombinant proteins, *B. subtilis* 168 derivatives (4088 *amyE*::pSHP1-6his*yjg*CD, 4233 *amyE*::pSHP2-*yjgC*8his, 4230 *amyE*::pSHP-6his*yrhED*, 4192 *amyE*::pSHP-*yrhE*8his) were grown in 4.8 L of the LB medium with 2 μM sodium molybdate at 37 °C until A_600_ = 0.3; cells were then submitted to a salt shock (0.4 M NaCl) for 30 min, followed by induction with 0.8% xylose for 3 h (4088 and 4233 strains) and expression overnight (4230 and 4192 strains) at 37 °C. Harvested cells were washed once in 20 mM sodium phosphate, pH 7.5, and 50 mM Na_2_SO_4_, and cells were kept at −80 °C until use.

### Plasmid construction

Genes were cloned into pSPH1 and pSPH2 to allow induction with the xylose promoter at the *amyE* locus. Plasmids were constructed as follows: the pET28HT-YjgCD: *yjgCD* fragment was amplified with primers 898-899 using *B. subtilis* gDNA as the template and inserted by ligation into a previously excised pET28dHT-*gapR* at *Nde*I-*Kpn*I sites to add a 6-his tag sequence followed by a TEV cleavage site at the 5′ end of *yjgC*.; pSPH1-6his*yjg*CD: 6his-*yjgCD* was amplified with primers 941 to 952 using pET28HT-YjgCD as the template and introduced by ligation into pSHP1 at *Avr*II*-Xho*I sites; pSPH1-6his*yrhED*: *yrhED* was amplified with primers 967 to 968 using *B. subtilis* 168 gDNA as the template and introduced into pSPH1 at *Avr*II-*Eco*RI sites using Gibson Assembly Cloning kit NEB with the linker primer 966. Cloning of *yrhE* at this site adds a sequence encoding a 6-his tag followed by a TEV cleavage site at the 5′ end. pSPH2-*yjgC*8his: *yjgC* was amplified with primers 944 to 950 and introduced into pSPH2 at *Xho*I-*Spe*I sites. Cloning of *yjgC* at this site adds a sequence encoding an 8-his tag at the 3′ end; pSPH1-*yrhE*8his: pSPH2-*yjgC*8his was digested at *Xho*I-*Sfi*I sites to release *yjgC* fragment. Then, a *yrhE* fragment was amplified with primers 988 to 1042 using *B. subtilis* gDNA as the template and introduced by ligation.

All plasmids were verified by sequencing, and primers are listed in Table S7.

### Protein purification

All steps were performed at 4 °C with an ÄKTA FPLC system (GE Healthcare). Frozen cells, typically 10 to 15 g of wet weight, were thawed and suspended in 90 ml of 20 mM sodium phosphate, pH 7.5, and 50 mM Na_2_SO_4_ (buffer A). Cell suspension was treated with protease inhibitor cocktail (Roche), lysozyme (1 mg/ml during 30 min at 37 °C), DNase I, and 0.5 mM EDTA and disrupted by 1 to 2 passages through a French pressure cell at 1 bar. Cells debris were removed by centrifugation for 40 min at 100,000*g*. The supernatant was applied onto 15 ml Ni-NTA affinity column (GE Healthcare). The column was washed with buffer A supplemented with 50 mM imidazole, and the enzyme eluted in buffer A supplemented with 500 mM imidazole and 10 mM KNO_3_. The enzyme was washed in 50 mM MES, pH 6, 50 mM Na_2_SO_4_, 5% glycerol, and 10 mM KNO_3_ using a PD-10 desalting column (Pharmacia), concentrated with Amicon 100 kDa (Millipore) and frozen in liquid nitrogen.

### Activity assays and kinetic analysis

FDH activity was determined using an AvaSpec-2048L spectrophotometer, inside an anaerobic chamber, with an atmosphere of 100% N_2_, at room temperature (RT), with stirring. For formate-BV oxidoreduction measurements, the reduction of BV was monitored at 600 nm (Ɛ_600 nm_ (BV^+^) = 7.0945 mM^−1^ cm^−1^) with 1.75 mM BV in 25 mM CHES, pH 8.6 (6his-YrhED), or 17.5 mM BV in 25 mM CHES, pH 10 (YjgC-8his/6his-YjgCD), supplemented with 50 mM sodium formate at RT. One unit of activity is defined as the amount of FDH capable of oxidizing 1 μmol of formate per minute per milligram of the enzyme. Kinetic analysis was measured at substrate concentrations ranging from 0.5 μM to 150 mM sodium formate. For formate:menadione oxidoreduction measurements, the reduction of 60 μM menadione was monitored at 260 nm (Ɛ_260 nm_ (menadione) = 17.2 mM^−1^ cm^−1^) in 25 mM CHES, pH 9, and 50 mM sodium formate at RT. One unit of activity is defined as the amount of FDH capable of reducing 1 μmol of menadione per minute per milligram of the enzyme. Kinetic analysis was measured at substrate concentrations ranging from 0.5 μM to 200 μM menadione. Kinetic parameters were calculated by direct fitting of the Michaelis–Menten equation: y = V_max_ × S/(K_M_ + S) using OriginPro. The influence of pH on FDH activity was assessed using a buffer mix containing 20 mM MES, CHAPS, CHES, and Hepes at RT. The pH was adjusted from 6.5 to 11 with HCl and NaOH.

### Quinone extraction and analysis

Quinones were extracted from solutions of purified ForCE1, ForCE2, or ForC1 essentially as described (66). Briefly, 20 to 50 μl of purified proteins (corresponding to 0.1–0.25 mg protein) was transferred into glass tubes and completed up to 200 μl with water. 50 μl KCl 3 M and 3 ml methanol were added, and the tubes were vortexed for 1 min. 2 ml petroleum ether (40–60° boiling range) was added, and vortex was repeated for 1 min. The tubes were centrifuged at 700 rpm for 1 min at RT, the upper phase was collected, and the methanol phase was extracted again with 2 ml petroleum ether. Both petroleum ether phases were combined in 5 ml Eppendorf tubes and dried under a nitrogen flow. Dried lipid extracts were resuspended in 100 μl ethanol, and fractions corresponding to 5 and 20 μg protein were analyzed by HPLC coupled to ECD and MS as previously described (67). The probe temperature was 400 °C, the cone voltage was 80 V, and MS spectra were recorded between *m/z* 500 and 900 with a scan time of 0.5 s.

Commercial MK-7 (Sigma) was used to generate standard curves ranging from 2 to 100 pmoles. The concentration of the MK-7 solution was determined using an extinction coefficient Ɛ_248 nm_ = 18,900 M^−1^ cm^−1^ (68). The standard curve for the ECD signal was used to quantify the peaks (MK-7 and MK-8) obtained for the protein samples. Technical duplicates (extraction and analysis) were performed for each protein preparation, and the quantifications of MKs represent the average of the four values obtained from two measurements (5 and 20 μg protein) for each duplicate.

### Redox titrations

#### Preparation of EPR samples

Formate-reduced samples of ForCE1 were prepared in 50 mM MES buffer, pH 6.0, with 80 μM of protein as followed. The air-oxidized sample was incubated with 100 mM sodium formate in a glove box and frozen immediately. Subsequently, the sample was thawed in the glove box and further reduced with 13 mM sodium dithionite before freezing. Formate-reduced samples of ForCE2 were prepared in 20 mM Tris/propane, pH 6.5, and 5% glycerol containing 50 μM of protein as followed. The air-oxidized sample was incubated with 30 mM sodium formate in a glove box before freezing or incubated with 5 mM sodium dithionite in a glove box before freezing.

Redox titrations of the MSK radical in ForCE1 were carried out anaerobically at RT (about 25 °C) either in a glove box or in an airtight vessel flushed with oxygen-free argon. Redox potentials were measured with a combined Pt-Ag/AgCl/KCl (3M) microelectrode and are given in the text with respect to the standard hydrogen electrode. The following redox mediators were used at 10 μM final concentrations: phenazine methosulfate, phenazine ethosulfate, methylene blue, resorufin, indigo carmine, anthraquinone 2,6 disulfate, flavin mononucleotide, phenosafranine, neutral red, and methyl viologen. Reductive titrations were carried out by stepwise addition of an anaerobic sodium dithionite solution (100 mM or 1 mM). Samples were anaerobically transferred into calibrated EPR tubes that were rapidly frozen.

The normalized variation of the peak-to-peak amplitude of the MSK EPR signal was fitted to a theoretical curve corresponding to two successive one-electron redox processes:

(1) $$A{\;}_{ptp}=\;\frac{1}{1+e^{\alpha \left(E-\;E_{1}\right)}+e^{\alpha \left(E_{2}-\;E\;\right)}}$$

Where E_1_ and E_2_ are the midpoint potentials of the MK/MSK and MSK/MKH_2_ couples, respectively. α = F/RT where R and F are the molar gas and Faraday constants, respectively, and T is the absolute temperature. The two-electron midpoint potential of the MK/MKH_2_ couple, which corresponds to the redox potential for which the amount of MSK is maximum, is E_m_ = (E_1_ + E_2_)/2. The stability constant K_S_ of MSK defined with respect to the comproportionation reaction is as follows:

(2) $$K_{S}=\frac{{\left[\mathrm{MSK}\right]}^{2}}{\left[MK\right]\left[MKH_{2}\right]}=e^{\alpha \left(E_{1}-E_{2}\right)}$$

The occupancy level R_occ_ was calculated as the ratio between the normalized MSK concentration per enzyme inferred from spin quantitation experiments (assuming one [2Fe-2S] cluster taken as internal reference per enzyme) and the normalized maximal MSK concentration (MSK_max_) given by the following equation.

(3) $$MSK_{\max}=\frac{1}{1+2e^{\mathrm{\alpha /}2\;\times \;\left(E_{2}-E_{1}\right)}}$$

Differences in the binding constants of MK (K_MK_) and MKH_2_ (K_MKH2_) manifest as a shift in the E_m_ from that of free MK/MKH_2_ given by the following equation:

(4) $$E_{m}\left(\mathrm{bound}\right)-E_{m}\left(\mathrm{free}\right)=\frac{1}{2}\alpha \;\times \;ln\left(\frac{K_{\mathrm{MK}H_{2}}}{K_{MK}}\right)\;$$

The affinity of the site for MSK is a determinant of E_1_ and E_2_ but not of E_m_.

#### Multifrequency EPR spectroscopy

X and Q band EPR spectra were measured on a Bruker Elexsys E500 spectrometer. For X band measurements, the spectrometer was equipped with an ER4012ST rectangular cavity fitted to an ESR900 helium flow cryostat (Oxford Instruments). For Q band measurements, a standard resonator equipped with an CF 935 cryostat (Oxford Instruments) was used. W band experiments were performed on a Bruker Elexsys E680 spectrometer equipped with a 6 T superconducting magnet and a 2-kG high-resolution sweep coil. Spectra were recorded with the standard W-band resonator fitted with a Bruker cryogen-free system (Stinger). For the echo-detected field-swept experiment, the two-pulse echo intensity was measured as a function of magnetic field at fixed time interval τ = 200 ns between the two microwave pulses, using a shot repetition time of 500 μs.

A field correction was applied to the magnetic field by simulating the overlapping spectral contribution of Mn^2+^, assuming g(Mn^2+^) = 2.00101 ± 0.00005 and an isotropic hyperfine coupling constant a(Mn^2+^) = −(8.710 ± 0.003) mT (69).

#### Spectral simulations

Numerical simulations of EPR spectra were performed with the EasySpin package (release 5.0.12) using MATLAB (The MathWorks, Inc) (70). A field-independent (unresolved hyperfine couplings, H-strain) linewidth model was used to simulate the EPR signal of the MSK and the Mo(V) species, whereas those of the FeS centers were simulated using a field-dependent (g-strain) linewidth model. For H-strain, the full width at half maximum of Gaussian lines along the g-tensor principal axes was adjusted, whereas the corresponding g-strain distributions were considered uncorrelated. Parameters used for the simulations shown in this work are provided in Table S8.

#### MS analysis

Purified proteins were subjected to an SDS-PAGE, and the stacking gel band corresponding to total proteins was excised and submitted to in-gel trypsin digestion for proteomic analysis as described previously (71) and with the following adapted modifications. Spectra were processed by Proteome Discoverer software (ThermoFisher, version 2.4.1.15) using the Sequest HT algorithm with the *Bacillus subtilis* database (Taxonomy ID: 224308, version 2016-08-20, downloaded from the NCBI by Protein Center including 5573 entries). In this study, proteins were also filtered by a minimum number of Peptide Spectral Match of 10. The list of identified proteins is available in Tables S2–S4.

## Data availability

The mass spectrometry proteomics data have been deposited to the ProteomeXchange Consortium (http://proteomecentral.proteomexchange.org) via the PRIDE partner repository (72) with the dataset identifier PXD028742 and 10.6019/PXD028742.

## Supporting information

This article contains supporting information (73, 74, 75, 76, 77, 78, 79, 80, 81, 82, 83, 84, 85, 86, 87).

## Conflict of interest

The authors declare that they have no conflicts of interest with the contents of this article.
