# Supplementary material for: Identification and characterization of a noncanonical menaquinone-linked formate dehydrogenase
Source: J Biol Chem. 2021 Nov 6;298(2):101384. doi: 10.1016/j.jbc.2021.101384 (PMC8808070; doi:10.1016/j.jbc.2021.101384)
Supplement: Figures S1–S7 and Tables S5–S8 [file mmc1.docx]

**Supporting information**

**Identification and characterization of a non-canonical menaquinone-linked formate dehydrogenase**

**Rodrigo Arias-Cartín, Alexandre Uzel, Farida Seduk, Guillaume Gerbaud, Fabien Pierrel, Marianne Broc, Régine Lebrun, Bruno Guigliarelli, Axel Magalon, Stéphane Grimaldi and Anne Walburger**

Supporting information contains Figures S1–S7, Tables S1-S9 and supporting Ref. 73-87

Figure S1 pS2

Figure S2 pS3-4

Figure S3 pS5

Figure S4 pS6

Figure S5 pS7

Figure S6 pS8

Figure S7 pS9

Table S1 pS10

Tables S2, S3, S4, S5 and S6 pS11

Tables S7, S8, S9 pS12

References pS13-14

**Figure S1.** **Phylogenetic tree and alignment of catalytic subunits of characterized Mo/W-*bis*PGD-containing FDH enzymes*.***

A. Pairwise sequence alignment of YjgC and YrhE from *B. subtilis* with predicted regions for [Fe-S] clusters C1 to C5 (orange) and Mo/W-*bis*PGD cofactor binding motif (grey). Position of amino acids involved in iron coordination are highlighted in red. Conserved amino acids between sequences are black-colored.

B. Full sequence alignment of 42 characterized FDH enzymes and of the two putative FDH proteins from *B. subtilis*. Alignments generated with Geneious Prime® 2020.1.1 (Global alignment: blosum62 matrix, Gap open penalty 12, Gap ext penalty 3 and 1000 refinement iterations, Tree building: Jukes-Cantor genetic distance model, neighbor joining method, no outgroup with resampling bootstrap and 1000 replicates). Protein sequences description is detailed in Table S1.

**
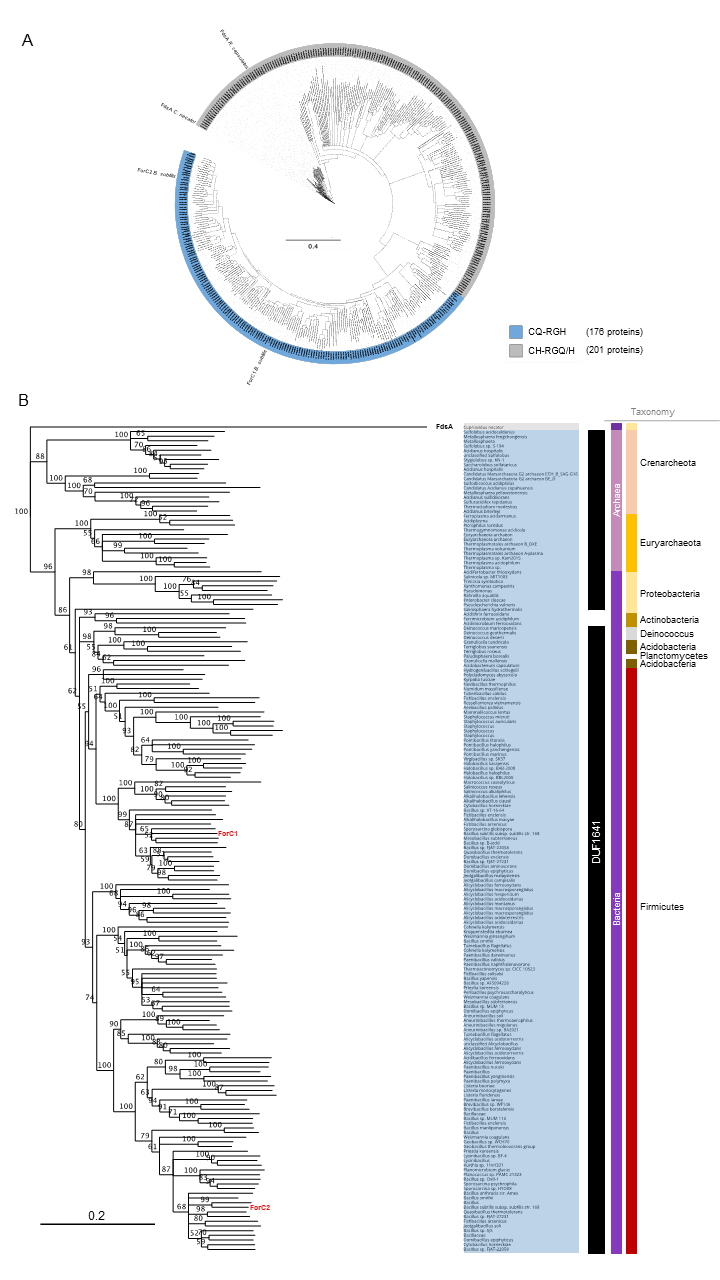
**

**Figure S2.** **Distribution of ForC and related proteins with the CQ RGH and CH RGQ/H motif.**

**A.** Phylogenetic tree of 377 sequences related to ForC and FdsA proteins and distribution of the CQ RGH and CH RGQ/H motif. The sequence dataset was obtained by merging the results of 3 independent BLAST searches, using the ForC1 sequence from *B. subtilis* as bait for the first search and the sequence with the lowest similarity score but displaying the CQ-RGH motif as a query for the other two consecutive searches. The results of each BLAST search were analysed with TREND (73) to remove sequence redundancy (CD-HIT: 0.9) and sequences lacking an N-terminal extension allowing coordination of 4 [Fe-S] clusters were removed. The final batch of sequences (377) was aligned and FdsA from *C. necator* was used as the root for phylogenetic tree construction. Sequences with a CQ RGH motif are colored in blue while the other are colored in grey.

**B.** Phylogenetic and taxonomy distribution of 176 sequences with the CQ RGH motif and the FsdA sequence from *C. necator*. The presence of a ForE (DUF1641) homolog in synteny with the ForC related protein was determined with TREND or by manual search. ForC1 and ForC2 from *B. subtilis* are highlighted in red, FsdA from *C. necator* is highlighted in black.

Alignments and phylogenetic trees were generated with Geneious Prime® 2020.1.1 (Global alignment: blosum62 matrix, Gap open penalty 12, Gap ext penalty 3 and 1000 refinement iterations, Tree building: Jukes-Cantor genetic distance model, neighbor joining method, outgroup FsdA from *C. necator* (WP_011614623) with resampling bootstrap and 1000 replicates). Phylogenetic tree in Fig S2A was edited using iTOL (74). The 377 sequence identifiers used for the phylogenetic tree are found in the Supplementary Information Table S9.


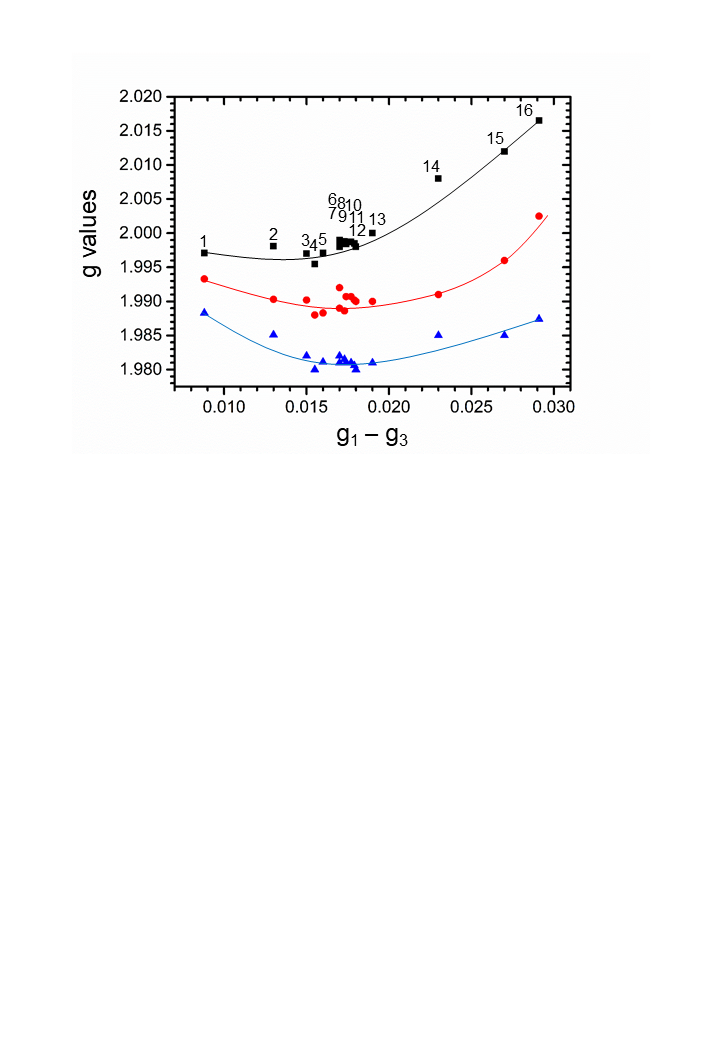


**Figure S3: Plot of the g values of Mo(V) species against g-tensor anisotropy for selected bacterial and eukaryotic Mo/W-*bis*PGD enzymes with Cys or SeCys ligand.** g_1_, g_2_ and g_3_ values are indicated by black squares, red circles and blue triangles, respectively. Nonlinear correlations can be extrapolated and are schematically indicated by solid lines. Data sets correspond to 1: *Bacillus subtilis* ForCE2 (this work), 2: *R. sphaeroides* dimethylsulfoxide reductase (S147C variant) (75), 3: *Synechococcus sp.* PCC 7942 assimilatory nitrate reductase NarB (76), 4: *Methanobacterium formicicum* FDH incubated with potassium cyanide (77), 5: *E. coli* periplasmic nitrate reductase NapA (78), 6: *Desulfovibrio desulfuricans* NapA (high g turnover Mo(V) species) (79), 7: *Azotobacter vinelandii* assimilatory nitrate reductase NAS (80), 8: *Paracoccus denitrificans* NapAB (81), 9: *R. sphaeroides* NapA (82), 10: *R. sphaeroides* NapAB (83), 11: *Shewanella gelidimarina* NapA (84), 12: *Paracoccus pantotrophus* NapAB (81), 13: *D. desulfuricans* NapA (high g nitrate Mo(V) species) (79), 14: *Paracoccus pantotrophus* NapAB incubated with thiocyanate (85), 15: *Desulfovibrio desulfuricans* FDH (86), 16: *Wolinella succinogenes* polysulfite reductase (87).


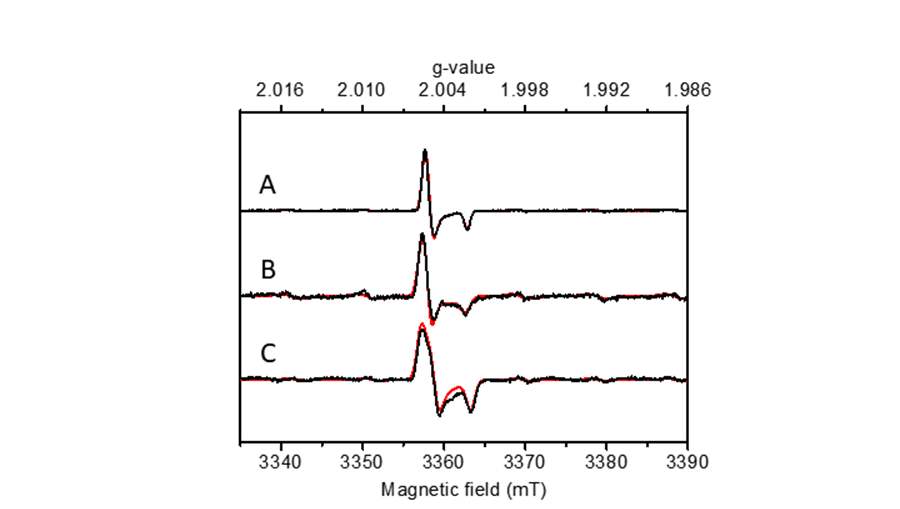


**Figure S4: W-band cw EPR spectra of MSK stabilized in ForCE1 (A), ForCE2 (B) and *Ec*NarGHI (C).** Simulations are shown in red and are superimposed to the experimental spectra shown in black. Simulation parameters are: g-tensor principal values g_1_,_2_,_3_ = 2.0054, 2.0051, 2.0023 (A), 2.0056, 2.0051, 2.0023 (B), 2.0058, 2.0048, 2.0022 (C). H-strain parameters (25.8, 25.8, 24.2) MHz (A), (33.6, 27.2, 38.1) MHz (B) and (34.4, 36.5, 34.2) MHz. (C). Experimental conditions: temperature, 50 K, microwave power, 0.05 mW (A, B) and 0.005 mW (C), modulation amplitude, 0.4 mT at 100 kHz (A, B) or at 10 kHz (C), microwave frequency, 94.2441 GHz (A), 94.0012 GHz (B) and 94.2493 GHz (C).

**

**

**Figure S5:** **pH dependence of *k*_cat_ (s^-1^) for formate:menadione oxidoreduction by ForCE2.**

**A**


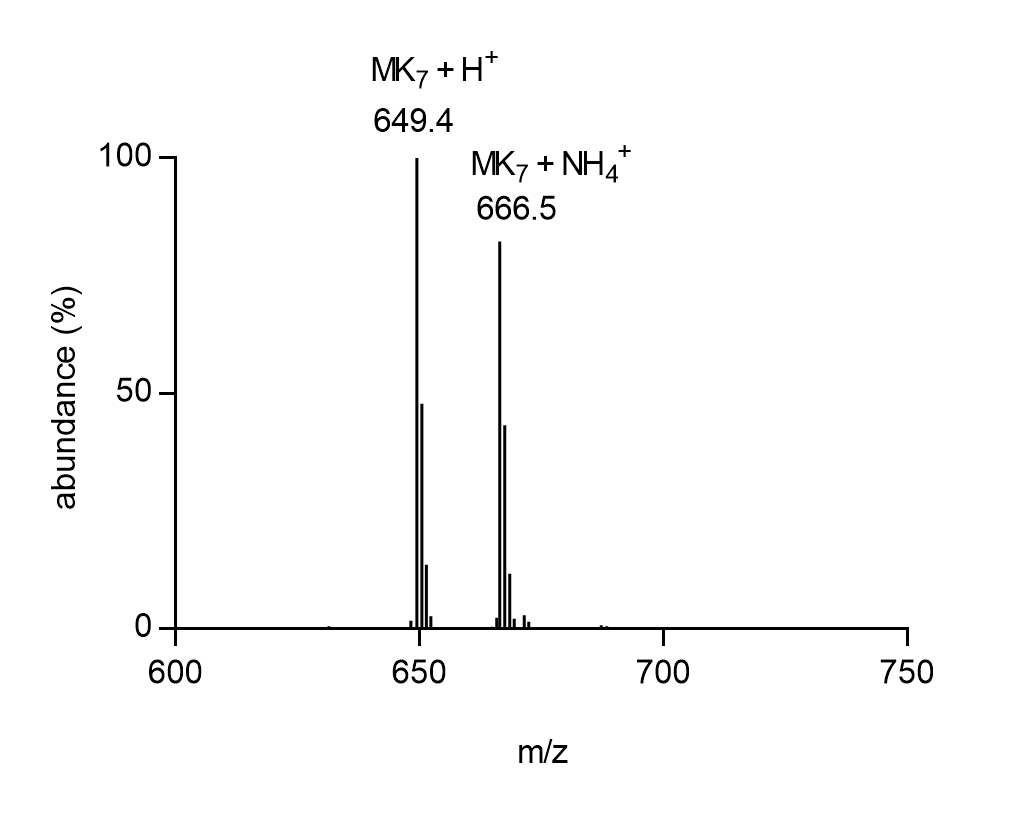


**B**


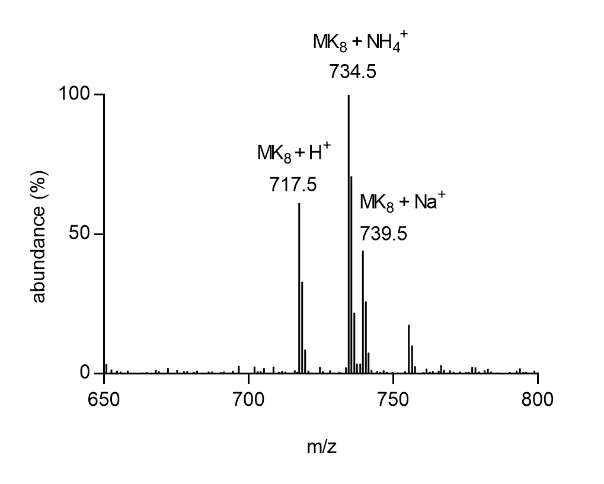


**Figure S6: Mass spectra of MK-7 and MK-8 by HPLC-MS**

A: Mass spectrum of MK-7 (eluting at 11.5 min) obtained by HPLC-MS analysis of lipid extracted from purified ForCE1.

B: Mass spectrum of MK-8 (eluting at 13.95 min) obtained by HPLC-MS analysis of lipid extracted from purified ForCE1.

**Figure S7. Amino acids surrounding the Mo/W atom in FDHs active site and their classification.** Mo/W (blue), ligands (yellow and white), *bis*-PGD (pink) and amino acids (cyan)

Class I type C/UH-RGQ: A. ForC1 model with CQ-RGH (based on 6TGA structure), B. FdsA from *R. capsulatus* (pdb: 6TGA) and C. FdhF from *E. coli* (pdb:1FDO)

Class II type UH-RGE: D. FdhA from *D. gigas* (pdb:1H0H) and E. FdhA from *D. vulgaris* (pdb: 6SDR)

Class III type UH-RGH: F. FwdBD from *M. wolfeii* (pdb: 5T5i) and G. FdnG from *E. coli* (pdb:1KQF).

**Table S1: FDH sequences used for alignments in figures 1A and S1B.**

| Protein Name | Organism | Identifier |
| --- | --- | --- |
| FmdB_METTF | *Methanothermobacter thermautotrophicus* | P95295 |
| FmdB_METTM | *Methanothermobacter marburgensis* | D9PXE7 |
| FmdB_METWO | *Methanothermobacter wolfeii* | O74033 |
| FmdB_METBA | *Methanosarcina barkeri* | Q48945 |
| FwdB_METKA | *Methanopyrus kandleri* | Q8TYN5 |
| FwdB_METTF | *Methanothermobacter thermautotrophicus* | Q59580 |
| FwdB_METWO | *Methanothermobacter wolfeii* | O74032 |
| FwdB_ARCFU | *Archaeoglobus fulgidus* | O28350 |
| FdhF_CORGL | *Corynebacterium glutamicum* | Q8NSY6 |
| FdhA_WOLSU | *Wolinella succinogenes* | Q7M971 |
| FdhA_ SULMU | *Sulfurospirillum multivorans* | Q6W8A3 |
| FdnG_DESVH | *Desulfovibrio vulgaris* | Q727P3 |
| FdhA_DESDE | *Desulfovibrio desulfuricans* | Q1KRL6 |
| FdhA_DESGI | *Desulfovibrio gigas* | Q934F5 |
| FdhA_DESAG | *Desulfovibrio alaskensis* | Q314X8 |
| FdhG_ PSEAE | *Pseudomonas aeruginosa* | Q14T72 |
| FdoG_ECOLI | *Escherichia coli (strain K12)* | P32176 |
| Fdh_CITS77 | *Citrobacter sp. S77* | WP_136345514 |
| FdnG_ECOLI | *Escherichia coli (strain K12)* | P24183 |
| YjgC_BACSU | *Bacillus subtilis (strain 168)* | O34720 |
| YrhE_ BACSU | *Bacillus subtilis (strain 168)* | Q795Y4 |
| FdsA_CUPOX | *Cupriavidus oxalaticus* | A0A4P7LDP8 |
| FdsA_CUPNE | *Cupriavidus necator* | O87815 |
| Fdh_RHOCB | *Rhodobacter capsulatus* | D5AQH0 |
| Fdh_METTR | *Methylosinus trichosporium OB3b* | A0A2D2D2X1 |
| FdsA_RHOPA | *Rhodopseudomonas palustris* | Q6NBU4 |
| Fdh1A_METEX | *Methylorubrum extorquens* | Q8KTI7 |
| Fdh_SYNFM | *Syntrophobacter fumaroxidans* | A0LP28 |
| Fdh_MOOTH | *FdhA Moorella thermoacetica* | P77908 |
| FdhAII_PEPAC | *Peptoclostridium acidaminophilum* | Q93V05 |
| FdhAI_PEPAC | *Peptoclostridium acidaminophilum* | Q93V06 |
| FdhF2_GOTA9 | *Gottschalkia acidurici* | K0B3A3 |
| FdhA_METFO | *Methanobacterium formicicum* | P06131 |
| FdhA_METTF | *Methanothermobacter thermautotrophicus* | Q50569 |
| FdhA2_METMI | *Methanococcus maripaludis* | Q877E3 |
| FdhA1_METMI | *Methanococcus maripaludis* | Q877E4 |
| Fdh_METVS | *Methanococcus vannielii* | A6UPU2 |
| FdhF_THEKI | *Thermoanaerobacter kivui* | A0A097ATK5 |
| FdhF_ECOLI | *Escherichia coli* | P07658 |
| FdhF1_ACEWD | *Acetobacterium woodii* | H6LB59 |
| FdhF2_ACEWD | *Acetobacterium woodii* | H6LB61 |
| FdhF_CLOPA | *Clostridium pasteurianum* | A0A0H3IZZ5 |
| FdhF_9CLOT | *Clostridium carboxidivorans P7* | C6PQ41 |
| FdhF_CLOLD | *Clostridium ljungdahlii* | D8GPQ2 |

Table heading for Tables S2-4: NCBI accession number; Protein name, name in NCBI; % of Coverage, percent protein sequence coverage by the matching peptides; Peptide Spectral Match, number of peptide spectral matches given by the Sequest algorithm corresponding to the total number of identified peptide sequences for the protein, including those redundantly identified; Unique peptides, number of distinct peptides matching to protein sequence and unique to this protein; MW (kDa), theoretical molecular mass of the identified protein (given by Sequest algorithm).

**Table S2: Mass spectrometry analysis of His-YjgCD/ForCE1 purification**

**Table S3: Mass spectrometry analysis of YjgC-His/ForC1 purification**

**Table S4: Mass spectrometry analysis of His-YrhED/ForCE2 purification**

| FDH | MK-7 (pmol/pmol of protein) | MK-8 (pmol/pmol of protein) |
| --- | --- | --- |
| ForCE1 | 0.87 ± 0.092 | 0.025 ± 0.009 |
| ForC1 | 0.016 ± 0.001 | ND |
| ForCE2 | 1.63 ± 0.82 | 0.035± 0.009 |

**Table S5: HPLC-ECD quantification of MK in lipid extracts of purified ForCE1, ForCE2 and ForC1 (n=2 independent purifications)**

**Table S6: Strains used in this work**

| Strains | Description | Reference |
| --- | --- | --- |
| 4161 | *Bacillus subtilis* 168 *trpC2* | BGSC |
| 4088 | *Bacillus subtilis* 168 *trpC2* *amyE*::pSHP1-6his*yjg*CD | This work |
| 4089 | *Bacillus subtilis* 168 *trpC2* *amyE*::pSHP2-*yjgC*8his | This work |
| 4040 | *Bacillus subtilis* 168 *trpC2* Δ*yjgD*::ery | This work |
| 4233 | *Bacillus subtilis* 168 *trpC2* Δ*yjgD*::*ery* *amyE*::pSHP2-*yjgC*8his | This work |
| 4156 | *Bacillus subtilis* 168 *trpC2* Δ*yjgC*:*:kan* | BGSC |
| 4167 | *Bacillus subtilis* 168 *trpC2* Δ*resE::ery* | BGSC |
| 4191 | *Bacillus subtilis* 168 *trpC2* Δ*resE::ery amyE::*pSHP1-6his*yrhED* | This work |
| 4192 | *Bacillus subtilis* 168 *trpC2* Δ*resE*::*ery* *amyE*::pSHP2-*yrhE*8his | This work |
| 4230 | *Bacillus subtilis* 168 *trpC2* Δ*resE*::*ery* Δ*yjgC*::*kan* *amyE*::pSHP1-6his*yrhED* | This work |

**Table S7: Primers used in this work**

| Primer | Sequence (5’ to 3’) |
| --- | --- |
| 898 | atttgtatttccaaggtcatatggctggcaagaaaacaatcacaa |
| 899 | tcgagtgcggccgcaagcttcaatctcgcttattcattcctctt |
| 941 | atgcatctagaaaggagattcctaggatggctcaccaccaccaccaccac |
| 944 | gatgggtaccgggccccccctcgagatggctggcaagaaaacaatcac |
| 952 | caagcttatcgataccgtcgacctcgagtcaatctcgcttattcattcc |
| 966 | atgacataaaatgcatctagaaaggagattcctaggatggctcaccaccaccaccaccacgaaaatttgtatttccaaggtcatatgatg |
| 967 | cgaaaatttgtatttccaaggtcatatgatggacgttaagtcgatcagtg |
| 968 | cgcggccgctctagaactagaattcttattgttctcgaagttcttttccc |
| 988 | gatgggtaccgggccccccctcgagatgatggacgttaagtcgatcagtg |
| 1042 | ggtggtggtggtggtggccattatggccatcagtcagatgtacatagccag |

**Table S8. Parameters used to simulate the EPR spectra shown in this work.**

| EPR-active species | Enzyme | Magnetic parameters | Linewidth parameters | Microwave frequency band |
| --- | --- | --- | --- | --- |
| Mo(V) | ForCE2 | g_1,2,3_ = 1.9971, 1.9934, 1.9890  ^1^A_1,2,3_ = 12.5, 12.4 & 11.0 MHz | 9.7, 12.0 & 8.9 MHz | X |
|  |  |  | 25.8, 20.7 & 19.4 MHz | Q |
|  |  |  | 82.7, 68.5 & 76.7 MHz | W |
| MSK | ForCE1 | g_1,2,3_ = 2.0054, 2.0051, 2.0023 | 28.5, 25.5 & 26.9 MHz | X |
|  |  |  | 25.8, 25.8 & 24.2 MHz | Q |
|  |  |  | 22.2, 28.4 & 21.1 MHz | W |
| [2Fe-2S]^1+^ | ForCE1 | g_12_, g_3_ = 2.0180, 1.9373 | 0.0085, 0.0116 | X |
| [2Fe-2S]^1+^ | ForCE2 | g_12_, g_3_ = 2.0203, 1.9357 | 0.0164, 0.0176 | X |
| [4Fe-4S]^1+^ | ForCE1 | g_1_,_2_,_3_ = 2.0474, 1.9484, 1.8966 | 0.0227, 0.0117, 0.0194 | X |
| [4Fe-4S]^1+^ | ForCE2 | g_1_,_2_,_3_ = 2.0295, 1.9401, 1.9119 | 0.0328, 0.6009, 0.0170 | X |

^1^ with colinear g- and proton hyperfine tensors

**Table S9: FDH identifiers used for the phylogenetic tree in Figure S2**

73. Gumerov, V. M., and Zhulin, I. B. (2020) TREND: a platform for exploring protein function in prokaryotes based on phylogenetic, domain architecture and gene neighborhood analyses. *Nucleic Acids Res*. 48, W72–W76

74. Letunic, I., and Bork, P. (2021) Interactive Tree Of Life (iTOL) v5: an online tool for phylogenetic tree display and annotation. *Nucleic Acids Res*. 49, W293–W296

75. George, G. N., Hilton, J., Temple, C., Prince, R. C., and Rajagopalan, K. V. (1999) Structure of the Molybdenum Site of Dimethyl Sulfoxide Reductase. *J. Am. Chem. Soc.* 121, 1256–1266

76. Jepson, B. J. N., Anderson, L. J., Rubio, L. M., Taylor, C. J., Butler, C. S., Flores, E., Herrero, A., Butt, J. N., and Richardson, D. J. (2004) Tuning a Nitrate Reductase for Function. *J. Biol. Chem.* 279, 32212–32218

77. Barber, M. J., May, H. D., and Ferry, J. G. (1986) Inactivation of Formate Dehydrogenase from *Methanobacterium formicicum* by Cyanide. *Biochemistry.* 25, 8150–8155

78. Jepson, B. J. N., Mohan, S., Clarke, T. A., Gates, A. J., Cole, J. A., Butler, C. S., Butt, J. N., Hemmings, A. M., and Richardson, D. J. (2007) Spectropotentiometric and Structural Analysis of the Periplasmic Nitrate Reductase from *Escherichia coli. J. Biol. Chem.* 282, 6425–6437

79. Najmudin, S., González, P. J., Trincão, J., Coelho, C., Mukhopadhyay, A., Cerqueira, N. M. F. S. A., Romão, C. C., Moura, I., Moura, J. J. G., Brondino, C. D., and Romão, M. J. (2008) Periplasmic nitrate reductase revisited: a sulfur atom completes the sixth coordination of the catalytic molybdenum. *J. Biol. Inorg. Chem.* 13, 737–753

80. Gangeswaran, R., Lowe, D. J., and Eady, R. R. (1993) Purification and characterization of the assimilatory nitrate reductase of *Azotobacter vinelandii. Biochem. J*. 289, 335–342

81. Butler, C. S., Charnock, J. M., Bennett, B., Sears, H. J., Reilly, A. J., Ferguson, S. J., Garner, C. D., Lowe, D. J., Thomson, A. J., Berks, B. C., and Richardson, D. J. (1999) Models for Molybdenum Coordination during the Catalytic Cycle of Periplasmic Nitrate Reductase from *Paracoccus denitrificans* Derived from EPR and EXAFS Spectroscopy. *Biochemistry*. 38, 9000–9012

82. Dementin, S., Arnoux, P., Frangioni, B., Grosse, S., Léger, C., Burlat, B., Guigliarelli, B., Sabaty, M., and Pignol, D. (2007) Access to the Active Site of Periplasmic Nitrate Reductase: Insights from Site-Directed Mutagenesis and Zinc Inhibition Studies. *Biochemistry.* 46, 9713–9721

83. Arnoux, P., Sabaty, M., Alric, J., Frangioni, B., Guigliarelli, B., Adriano, J.-M., and Pignol, D. (2003) Structural and redox plasticity in the heterodimeric periplasmic nitrate reductase. *Nat. Struct. Biol.* 10, 928–934

84. Simpson, P. J. L., McKinzie, A. A., and Codd, R. (2010) Resolution of two native monomeric 90kDa nitrate reductase active proteins from *Shewanella gelidimarina* and the sequence of two *napA* genes. *Biochem. Biophys. Res. Commun.* 398, 13–18

85. Butler, C. S., Charnock, J. M., Garner, C. D., Thomson, A. J., Ferguson, S. J., Berks, B. C., and Richardson, D. J. (2000) Thiocyanate binding to the molybdenum centre of the periplasmic nitrate reductase from *Paracoccus pantotrophus.* *Biochem J.* 352,859-64.

86. Rivas, M. G., González, P. J., Brondino, C. D., Moura, J. J. G., and Moura, I. (2007) EPR characterization of the molybdenum(V) forms of formate dehydrogenase from *Desulfovibrio desulfuricans* ATCC 27774 upon formate reduction. *J. Inorg. Biochem.* 101, 1617–1622

87. Prisner, T., Lyubenova, S., Atabay, Y., MacMillan, F., Kröger, A., and Klimmek, O. (2003) Multifrequency cw-EPR investigation of the catalytic molybdenum cofactor of polysulfide reductase from *Wolinella succinogenes*. *J. Biol. Inorg. Chem*. 8, 419–426
